# Anti-Biofilm Effect of Hybrid Nanocomposite Functionalized with Erythrosine B on *Staphylococcus aureus* Due to Photodynamic Inactivation

**DOI:** 10.3390/molecules29163917

**Published:** 2024-08-19

**Authors:** Larysa Bugyna, Katarína Bilská, Peter Boháč, Marek Pribus, Juraj Bujdák, Helena Bujdáková

**Affiliations:** 1 Department of Microbiology and Virology, Faculty of Natural Sciences, Comenius University in Bratislava, Ilkovičova 6, 842 15 Bratislava, Slovakia; larysa.bugyna@uniba.sk (L.B.); bilska6@uniba.sk (K.B.); 2 Institute of Inorganic Chemistry, Slovak Academy of Sciences, Dúbravská Cesta 9, 845 36 Bratislava, Slovakia; peter.bohac@savba.sk (P.B.); marek.pribus@savba.sk (M.P.); juraj.bujdak@uniba.sk (J.B.); 3 Department of Physical and Theoretical Chemistry, Faculty of Natural Sciences, Comenius University in Bratislava, Ilkovičova 6, 842 15 Bratislava, Slovakia

**Keywords:** *S. aureus*, erythrosine B, photosensitizer, nanocomposite, photodynamic inactivation

## Abstract

Resistant biofilms formed by *Staphylococcus aureus* on medical devices pose a constant medical threat. A promising alternative to tackle this problem is photodynamic inactivation (PDI). This study focuses on a polyurethane (PU) material with an antimicrobial surface consisting of a composite based on silicate, polycation, and erythrosine B (EryB). The composite was characterized using X-ray diffraction and spectroscopy methods. Anti-biofilm effectiveness was determined after PDI by calculation of CFU mL^−1^. The liquid PU precursors penetrated a thin silicate film resulting in effective binding of the PU/silicate composite and the PU bulk phases. The incorporation of EryB into the composite matrix did not significantly alter the spectral properties or photoactivity of the dye. A green LED lamp and laser were used for PDI, while irradiation was performed for different periods. Preliminary experiments with EryB solutions on planktonic cells and biofilms optimized the conditions for PDI on the nanocomposite materials. Significant eradication of *S. aureus* biofilm on the composite surface was achieved by irradiation with an LED lamp and laser for 1.5 h and 10 min, respectively, resulting in a 10,000-fold reduction in biofilm growth. These results demonstrate potential for the development of antimicrobial polymer surfaces for modification of medical materials and devices.

## 1. Introduction

*Staphylococcus aureus* is the most well-known and widespread Gram-positive bacterium causing a variety of bacterial infections, such as superficial skin and soft tissue infections, and also contributes to severe systemic diseases [1,2,3,4,5,6,7]. The emergence of antibiotic-resistant strains, in particular, methicillin-resistant *S. aureus* (MRSA) [8,9,10,11], created a serious problem in outpatient, surgical, and hospital settings that requires research into new alternative antimicrobial approaches [12]. One of major problems is the biofilms formed by *S. aureus* on surfaces [13,14,15] protected by an extrapolymeric substance, making them highly resistant to antibiotics and the host immune response [15,16,17,18,19]. Biofilms contribute to the persistence of infection, complication of treatment, and the risk of relapse [20]. Some nanomaterials are used to prevent or reduce the biofilm formation [21,22,23,24,25,26]. Over the years, various conventional approaches were used for disinfection of different surfaces. In medicine, these include catheters, implants, prostheses, medical devices, water supply systems, filters, many industrial devices, and household surfaces [21,27,28,29,30,31,32,33]. In any case, each of these surfaces has its weakness in terms of efficacy, longevity, and stability [34,35]. Dadi et al. demonstrated that polyurethane (PU) modified with organoclay based on layered silicate saponite (Sap) functionalized with a photosensitizer (PS) was effective against biofilm of *S. aureus* after photodynamic inactivation (PDI) [36,37].

The PDI is an innovative strategy originally developed for the treatment of various cancers or the skin and oral cavity infections [38,39,40]. This approach uses light-activated PS followed by the formation of reactive oxygen species (ROS) [41,42,43], resulting in the subsequent death of the microbial cell. PDI offers many advantages over conventional antimicrobial treatments, including no resistance development and wide potential for localized therapy [28,44,45,46,47].

In 1876, the British chemist Kussamaul [48] first synthesized the red xanthene dye erythrosine B (EryB) [49,50,51,52], which became a promising target for photodynamic therapy (PDT) [53,54,55,56,57,58] as a potential PS [52,59,60,61,62] against Gram-negative and Gram-positive bacteria [54,63,64,65,66,67]. It demonstrated favorable photophysical properties, including high absorption in the visible spectrum and efficient generation of ROS upon light activation. Upon light activation, EryB undergoes photochemical reactions leading to the generation of ROS, including singlet oxygen (^1^O_2_) [68,69], which promote antimicrobial activity by destroying proteins, nucleic acids, and bacterial cell membranes, and ultimately leading to the death of microbial cells [69,70].

Only a few studies demonstrated some antimicrobial efficacy of EryB-based PDI against Gram-positive bacteria, including *S. aureus* [40,65]. In this study, EryB was used to study the effectiveness of a nanocomposite prepared using a similar approach previously described for phloxine B in research of Dadi et al. against *S. aureus* biofilm [36,37]. In addition, various factors, such as the periods of irradiation and the irradiation sources (green LED lamp vs. green laser), were compared regarding their impact on the PDI efficiency. This work is a pilot study to introduce a hybrid nanocomposite with functionalized EryB that could be used to modify polymers in the medical industry.

## 2. Results and Discussion

### 2.1. Physico-Chemical Characterization of Nanocomposite

**Figure 1:**
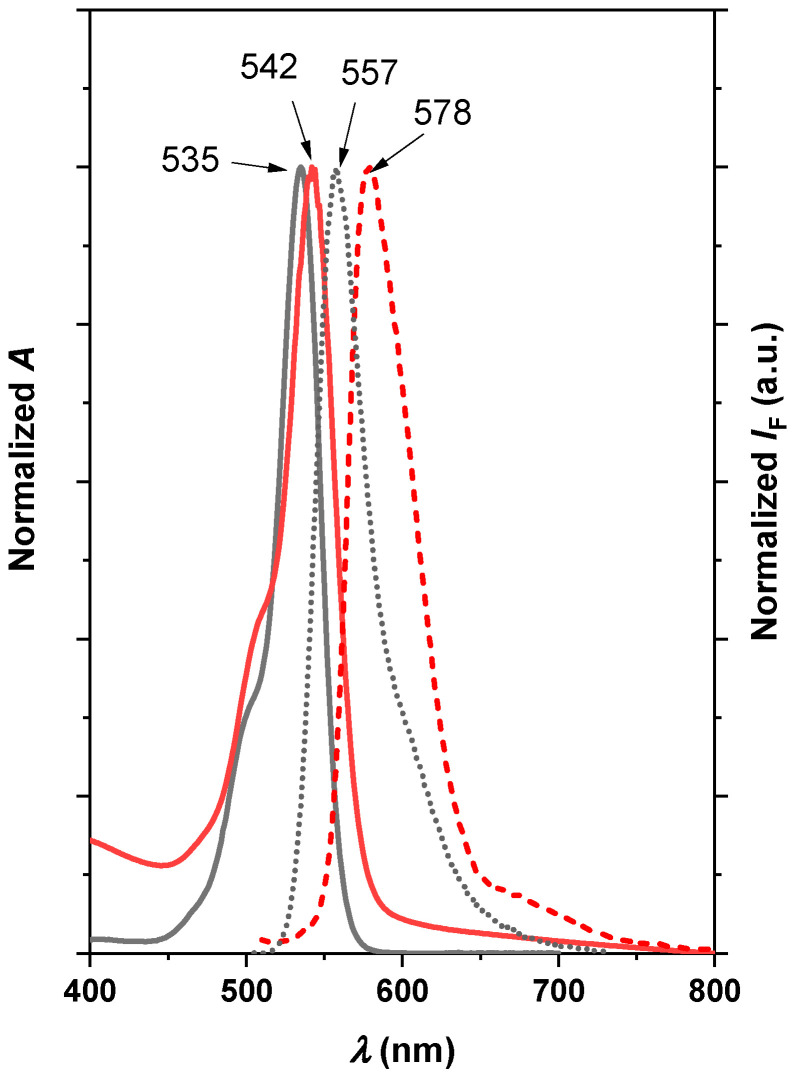
Normalized absorption (solid lines) and fluorescence spectra (dotted lines) of EryB solution (grey) and the nanocomposite films (red). The fluorescence spectra were recorded using the excitation wavelength at 490 nm.

Normalized absorption and fluorescence spectra of EryB solution and the nanocomposite films are shown in Figure 1. EryB solutions exhibited an absorption maximum at 535 nm, with a 0–1 vibronic shoulder at 497 nm. Adsorption of EryB on a Sap/PDDA nanocomposite film led to a shift in the main band to a longer wavelength with respect to the solution (from 535 to 542 nm) with a relatively more intense shoulder at 505 nm, which could be attributed to the formation of molecular H-dimers. A similar behavior was observed in the fluorescence spectra, where we observed a shift in the main emission band of the ethanol solution of EryB from 557 nm to 578 nm of the nanocomposite film at the same excitation wavelength of 490 nm. The observed redshift can be attributed to a more hydrophobic environment in nanocomposite film than in aqueous solution [71]. The preparation of the nanocomposite did not lead to significant molecular aggregation of the EryB dye, so the fluorescence and photoactivity of the dye remained at a standard level [72].

**Figure 2:**
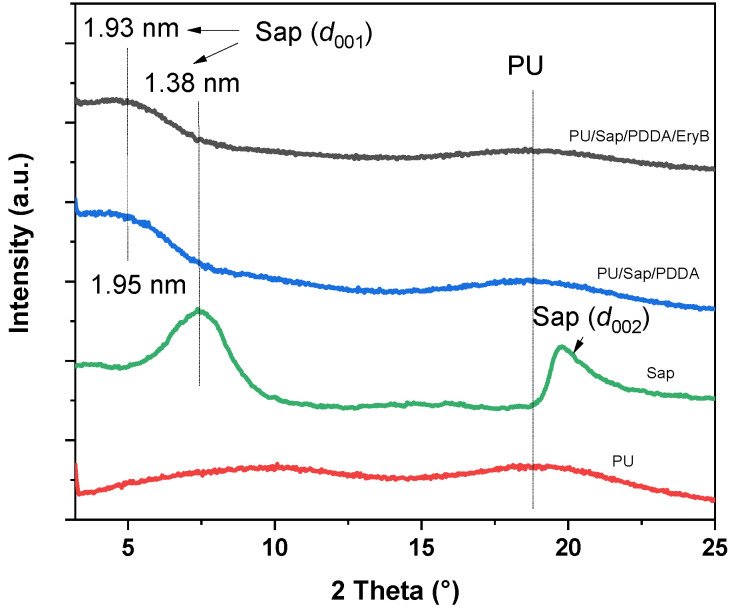
X-ray diffraction patterns of raw Sap, PU, and PU nanocomposites of PU/Sap/PDDA and PU/Sap/PDDA/EryB. The numbers labeling the reflection peaks show d values (nm).

The expansion of the interlayer spaces of Sap by intercalation with polycation, dye, or polymer can be detected by X-ray diffraction (XRD, Figure 2). The polymer composites were compared with unmodified Sap. The expansion of the basal spacing *d*_001_ increased from 1.38 nm in Sap to about 1.95 nm in PU/Sap/PDDA and 1.93 nm in PU/Sap/PDDA/EryB. This change can be assigned to the intercalation of Sap layers, exchanging hydrated exchangeable cations and free water molecules. The addition of EryB had no effect on the resulting value of the basal spacing. The semi-crystalline PU phase can be detected in the range of 15°−25° (2*θ*). The reflections assigned to the PU phase did not change significantly in the XRD patterns of the composite materials. The detection of the polymer phase in the composites may be caused by the actual presence of PU in the organoclay composites, but a large penetration depth of X-rays beneath the films reaching the pure polymer phase should also be considered. The polymer chains most likely did not intercalate in the interlayer spaces of the organoclay phase. The intercalation would lead to a more pronounced expansion of the interlayer spaces and a shift of the basal reflections to a much lower angle.

**Figure 3:**
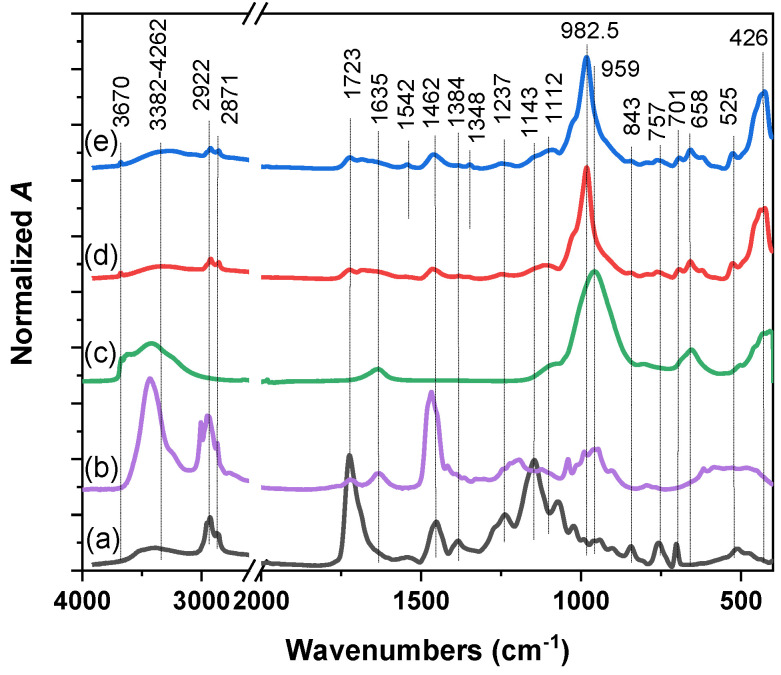
ATR IR spectra of the precursors (a) PU, (b) PDDA, (c) Sap, and composites (d) PU/Sap/PDDA, and (e) PU/Sap/PDDA/EryB.

Attenuated total reflectance infrared (ATR IR) spectroscopy was performed to detect the presence of individual components and functional groups on the surface of the prepared samples. The bands assigned to the vibrations of characteristic groups of both the Sap and organic phases of the components can be found in the spectra of nanocomposites (Figure 3). Their identification was confirmed by comparison with the spectra of the individual components or precursors.

The presence of H_2_O is reflected in the spectra of Sap (Figure 3c) by a broad complex band near 3440 cm^−1^ and 1635 cm^−1^. After modification with organic cations, most of the water molecules were removed from the samples, indicating a hydrophobic environment. This process decreased the overall intensity of νOH from H_2_O (Figure 3d,e). Two bands at 2922 and 2871 cm^−1^ were attributed to the C-H stretching vibrations of PU and PDDA cations, respectively. Corresponding C-H bending vibrations were present between 1500 and 1300 cm^−1^ [73]. An intense band at a wavenumber of 959 cm^−1^ indicates the Si-O stretching vibration of the Sap component, which is shifted to 982.5 cm^−^^1^ in the prepared nanocomposites. The bands at 658 and 426 cm^−1^ correspond to the bending vibrations of the structural OH (MgMgMg-OH) and Si-O-Mg bonds present in Sap layers and the band at 1112 cm^−^^1^ to the stretching Si-O vibrations of the basal O. A characteristic band of the PU was a strong band at 1723.5 cm^−1^ corresponding to the C=O bond vibrations [74]. A bending vibration band of C-H of the organic compounds was detected at 762 cm^−^^1^. The presence of these bands in the spectra of nanocomposites is evidence of diffusion of the polymer to the surface of the composites [75]. Other characteristic bands of PU at 1145 cm^−1^ corresponding to the stretching vibrations of C-O-C, at 1072 cm^−1^ to the stretching vibrations of C-O, and complex vibrations in the range of 1200–1450 cm^−1^ of C-H, N-H were not detected in the nanocomposite samples [76]. EryB can be indicated by indistinct bands at 1542 and 1348 cm^−1^, which can be assigned to C-H and N-H vibrations, respectively.

### 2.2. Photosensitizing Properties of the Nanocomposites

**Figure 4:**
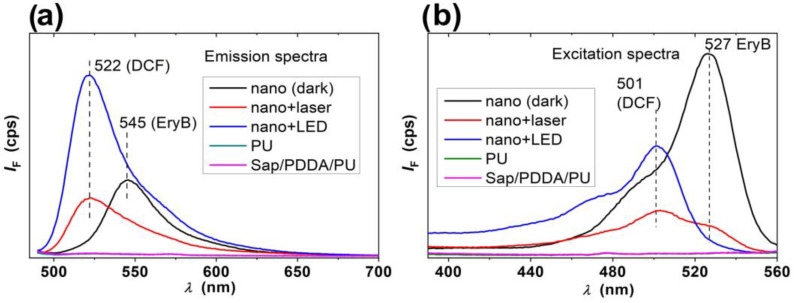
Emission (**a**) and excitation spectra (**b**) to identify the species formed after irradiation of the samples in DCFH/phosphate buffer solution. Nano stands for the nanocomposite samples with EryB. PU and Sap/PDDA/PU are control samples without EryB. The dashed lines show the positions of the respective wavelengths to indicate the bands characteristic of DCF or EryB species. The green line is overlapped with pink line.

The buffer solution of 2,7-dichlorodihydrofluorescein diacetate (DCFH DA) was hydrolyzed in a basic phosphate buffer solution to obtain an active probe, 2,7-dichlorodihydrofluorescein (DCFH) [77]. This compound can detect ROS [78,79,80], which was formed from molecular oxygen by photosensitization by EryB molecules on the surface of the functionalized composites. The ROS reacted with DCFH and formed strongly fluorescent 2,7-dichlorofluorescein (DCF) with maximum emission at about 522 nm. The emission spectra in Figure 4a confirm the formation of ROS in the presence of nanomaterials containing EryB and irradiated with laser or LED. The stronger emission at 522 nm after the LED irradiation can be explained by the longer treatment time. The excitation spectra (Figure 4b) confirmed this trend and detected a DCF band with a maximum at 501 nm. Blank samples of the same nanocomposite treated in the dark, PU without modification, and PU composite without EryB exhibited no ROS formation. This is evidence that ROS formation is related to the photosensitizing properties of EryB, which is in accordance with other studies and medical applications [81,82,83,84].

However, in addition to DCF, EryB molecules released from the nanocomposites were detected in some samples, which was proved by the bands with maximums at 527 and 545 nm in the excitation and emission spectra, respectively. The largest amount of EryB was detected in the sample treated in the dark. A much smaller amount was detected in the excitation spectrum of the composite irradiated with the laser and no EryB was detected in the spectra of the other samples. This was to be expected for the control samples PU and Sap/PDDA/PU composite. However, EryB was also absent in the solution of an LED-treated composite sample functionalized with dye. EryB was likely released from the composites independently of the irradiation, but the released EryB molecules were probably decomposed by the reactions with ROS. 

In summary, the experiments to detect the species formed during irradiation demonstrated the photosensitizing properties of the EryB-containing composites. The release of EryB from the composites was also demonstrated, which is an essential property for efficient PDI, especially for the reduction in biofilm growth. Without the release of EryB, biofilm growth would not be efficiently reduced and the PDI effect would be limited to much fewer photoactive EryB molecules at the interface. The photosensitizer was partially or completely degraded during irradiation, confirming a high reactivity of the ROS formed during photosensitization. This explains why it is so important to prepare modified surfaces with a high concentration of photosensitizer to achieve the desired effects in the irradiation of biofilms.

### 2.3. Antimicrobial and Anti-Biofilm Activity of PDI

*S. aureus* is one of the most dangerous human pathogens responsible for numerous nosocomial infections [6]. The widespread use of antibiotics against this bacterium led to a major problem in terms of antibiotic resistance [13]. Therefore, new alternative approaches are needed to inactivate and treat this pathogen. The PDI technique showed good results in inactivating *S. aureus* and does not lead to resistance [57]. Skoura et al. demonstrated in their research that when different concentrations of phloxine B are integrated into nanocomposites, a positive antibacterial effect occurs under the action of PDI [85]. In addition, Dadi’s work showed increased effectiveness of PDI by 10,000-fold with nanomaterials functionalized with PhB against CCM 3953 using a green laser [36]. For this reason, we were interested in testing another xanthene dye, EryB, and demonstrating its photoactive properties in combination with nanocomposites against *S. aureus*.

**Figure 5:**
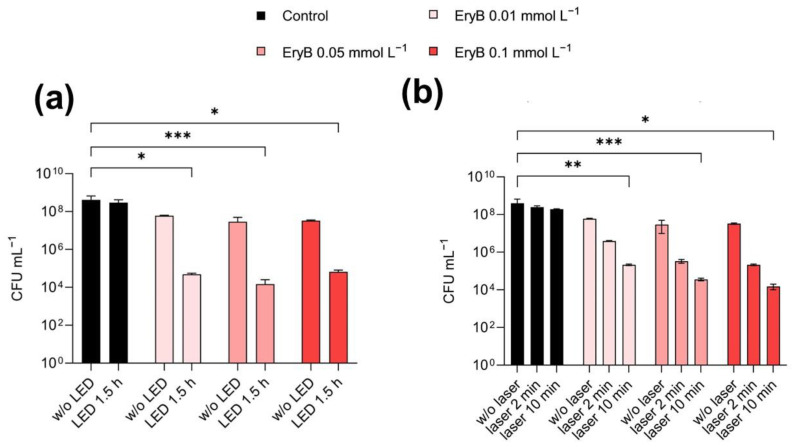
The antimicrobial effectiveness of EryB against *S. aureus* CCM 3953 without and after irradiation with a green LED light and green laser. Samples were cultured in the presence of different concentrations of EryB with a 1 h pre-incubation; (**a**) samples irradiated with a green LED lamp, and (**b**) samples irradiated with a green laser. A *p* < 0.05 (*) was considered significant; *p* < 0.01 (**) and *p* < 0.001 (***).

In the preliminary experiments, the effectiveness of EryB was tested on planktonic cells in three different concentrations, namely 0.01 mmol L^−^^1^, 0.05 mmol L^−^^1^, and 0.1 mmol L^−^^1^. The samples were irradiated with a green LED lamp and a green laser with different irradiation periods to compare effects of irradiation of both light sources. Figure 5 shows a slight inhibitory effect of EryB alone on planktonic cells, while after irradiation, the growth was inhibited 100-fold compared to the control samples without EryB. Samples with EryB at concentrations of 0.05 mmol L^−^^1^ and 0.1 mmol L^−^^1^ showed the highest inhibitory effect when reduced the survival of planktonic cells by 10,000-fold irradiated with both green laser (10 min) and green LED light for 1.5 h compared to the control samples representing growth without EryB. 

Based on the results of the test with planktonic cells, 0.05 mmol L^−^^1^ and 0.1 mmol L^−^^1^ concentrations were tested to know if EryB is also effective against biofilm. There was used only one period of irradiation: 1.5 h and 10 min for a green LED lamp and a green laser, respectively. This preliminary experiment was performed in microtiter plates and was supposed to help set the conditions for the main experiment focused on anti-biofim effectiveness on PU-modified discs. Results summarized in Supplementary data (Figure S1) show that anti-biofilm efficacy of tested concentrations of EryB resulted to an approximately 10,000-fold decrease in biofilm growth when the samples were irradiated with green LED light for 1.5 h, but growth in biofilm cells decreased by 100-fold for to the samples irradiated with a green laser for 10 min. The samples with a concentration of 0.1 mmol L^−^^1^ after irradiation showed only a slightly higher inhibition compared to the samples with 0.05 mmol L^−^^1^ of EryB. Similar effectiveness of EryB against *S. aureus* biofilm was also confirmed by Silva et al. when 0.5 mmol L^−1^ of EryB after 30 min pre-incubation in the dark and 30 min irradiation with green LED light eradicated biofilm. Moreover, they observed significant disruption of biofilm architecture after PDI by scanning electron microscopy [63]. As can be seen from the presented results, the light source and the duration of irradiation also play key roles. The combination of the power of the irradiation device, the duration of irradiation, and the size of the irradiated area is important for the overall effectiveness of the irradiation. It is therefore particularly important to match these conditions optimally to the type of experiment. Both lasers and LED lamps are frequently used as light sources in PDI. However, clinical studies showed that LED lamps are used less frequently than lasers. The advantage of lasers is that they can deliver more energy than other sources, but this energy is limited to a small area. In addition, lasers have a narrow emission bandwidth, so the specific absorption peak of PS can be targeted. The limitations of lasers can be potential eye safety issues and their price. LEDs can be used for larger treatment areas, but the limitations are size, the fact that they are cumbersome, and potential heat production. What is important is achieving uniform dispersion of LED light [86,87,88]. 

### 2.4. Anti-Biofilm Effectiveness of PDI with Nanocomposite

In recent years, studies showed the potential of photoactive composites to have an antibacterial effect. Zmejkoski et al. synthesized photoactive nanochitosan dots that were encapsulated in a bacterial cellulose polymer matrix and exhibited high anti-biofilm properties against MRSA after irradiation with a green laser [89]. The study of Xu et al. introduced photoactive Ag-nanoparticles containing PSs chlorin e6 and 4-mercaptobenzonitrile. The results show antibacterial effectiveness against *S. aureus* more than 99% inhibition of colony growth after photo-activated release of ROS and Ag^+^ with a laser (655 nm) [90]. Bilská et al. confirmed the effectiveness of a photoactive nanocomposite system contains PS phloxine B against MRSA enhanced by production of ROS after irradiation with a green laser [43]. In the study of Garapati et al., EryB nanoparticles based on poly-lacticco-glycolic acid were tested against *S. aureus* for potential use in the treatment of sinusitis. Similar to our work, they also observed slow release of EryB from particles and a significantly higher effect of EryB nanoparticles after PDI compared to the effectiveness of EryB alone [53].

**Figure 6:**
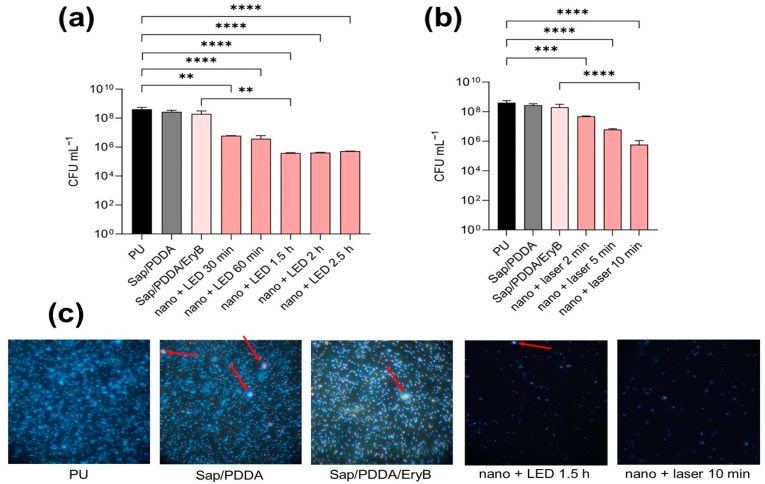
The anti-biofilm effectiveness of nanocomposite against 24 h biofilm of *S. aureus* CCM 3953; (**a**) PDI after irradiation with green LED light; (**b**) PDI after irradiation with a green laser; nano represents PU/Sap/PDDA/EryB discs; (**c**) fluorescence microscopy of biofilm cells isolated from different samples stained with DAPI, magnification was 1000×, nano represents PU/Sap/PDDA/EryB discs; red arrows mark particles of organoclay or organoclay modified with EryB. A *p* < 0.01 (**) were considered significant; and *p* < 0.01 (**), *p* < 0.001 (***), and *p* < 0.0001 (****) extremely significant.

In this study, the nanocomposites were tested according to the protocol of Dadi et al. [36,37]. Figure 6 demonstrates the growth of the staphylococcal biofilm compared to the control sample of unmodified PU and the modified nanocomposites PU/Sap/PDDA and PU/Sap/PDDA/EryB. Results are supported by examination in fluorescence microscopy. Anti-biofilm efficacy was not observed in the case of the non-irradiated samples. Green LED light was used to irradiate the samples for several periods: from 30 min to the maximum irradiation time of 2.5 h. An irradiation period of 1.5 h gave the best results in terms of anti-biofilm activity reducing growth by up to 10,000-fold. Longer irradiation periods did not lead to a significant increase in efficacy (Figure 6a). Compared to PDI with a green laser, an irradiation time of 10 min was sufficient to achieve the same effect as irradiation with a LED lamp for 1.5 h. Irradiation for 2 and 5 min resulted in reduction in biofilm growth by 100-fold and 1000-fold, respectively (Figure 6b). Figure 6c documents density of biofilm cells isolated from control samples and selected irradiated and non-irradiated nanomaterial with EryB. Results agree with a trend in reduction in biofilm cells described in Figure 6a,b.

The results show a significant effect of PDI with both light sources, which is consistent with other studies [91,92]. This confirms that the combination of concentration of PS, the light source, and the duration of light exposure is crucial for the effectiveness of nanomaterial with EryB. This study may help to classify PDI with EryB as a potential clinical application.

## 3. Materials and Methods

### 3.1. Bacterial Strains, PS, and Light Source

*S. aureus* CCM3953 (Czech Collection of Microorganisms, Brno, Czech Republic) was used for experimental research. Strain was retrieved from stock sample kept at −20 °C and incubated overnight at 37 °C in Mueller–Hinton broth (MHB; Biolife, Milan, Italy). After 16 h, the culture was transferred into fresh MHB and grown for approximately 2.5 h (exponential phase OD_600_ = 0.5; corresponding to 2 × 10^7^ cells mL^−1^) [85] at 37 °C with shaking at 150 rpm in an incubator (Thermo shaker PST-60H2-4 Biosan, Riga, Latvia). This culture was used for the tests.

EryB (Sigma Aldrich Corporation, St. Louis, MO, USA) was dissolved in 20 mL of H_2_O, sonicated (Branson 200 ultrasonic cleaner, Danbury, CT, USA), and sterilized by filtration through 0.22 μm pore diameter membranes (TPP, Trasadingen, Switzerland). The dye solution was stored in the dark at 4 °C. The absorbance at 530 nm was measured using a UV-Vis spectrophotometer (1 mL in cuvette, BOECO, Hamburg, Germany). The antimicrobial properties of EryB were tested in preliminary experiments with different concentrations (0.01 mmol L^−^^1^, 0.05 mmol L^−^^1^, and 0.1 mmol L^−^^1^).

Two different light sources and different periods of irradiation were used to compare the effects. Irradiation with a green laser (*λ* = 532 nm, 100 mW, Alligator, MZTech s.r.o., Košice, Slovakia) was set to 2 min, 5 min, and 10 min, and irradiation with a green light-emitting diode (LED) (2.42 mW cm^−^^2^, Weeylite, Sprite 20 RGB, Guangdong, China) was taken for 30 min, 60 min, 1.5 h, 2 h, and 2.5 h. The spectrum of the LED light source was obtained by a spectrophotometer C-700R spectromaster (Sekonic, Tokyo, Japan) and it is shown in Supplementary data (Figure S2). The distance between the laser tip or the LED lamp and the bottom of the 24-well plate (TC Plate, Sardstedt AG & Co, Nümbrecht, Germany) was set to 5 and 6 cm, respectively, during irradiation. This distance ensured that the samples were not exposed to significant temperature changes, preventing overheating and possible cell death. To prevent dehydration during subsequent irradiation, 50 μL of phosphate-buffered saline (PBS; 137 mM NaCl, 2.7 mM KCl, 8 mM Na_2_HPO_4_, and 2 mM KH_2_PO_4_; CentralChem, Bratislava, Slovakia) was added to the wells designated for laser irradiation, while 250 μL of PBS was added to those designated for LED lamp irradiation.

### 3.2. Preparation of Nanocomposites

To test the antimicrobial properties PU (VARNISH-PU 2KW, Isomat S.A., Thessaloniki, Greece), discs were prepared, the surface of which was modified with EryB containing clay–polymer nanocomposites.

A synthetic layered silicate saponite Sumecton (Sap) with the structural formula (Na_0.49_Mg_0.14_)^+0.77^[(Si_7.20_Al_0.80_)(Mg_5.97_Al_0.03_)O_20_(OH)_4_]^–0.77^ and cation exchange capacity CEC of 0.87 ± 0.05 mmol⋅g^–1^ [93] (Kunimine Industries Co., Ltd., Tokyo, Japan) was used as an inorganic carrier of PS. A stock dispersion of Sap was prepared with a concentration of 4 g L^−^^1^ by dispersing Sap in MilliQ water. It was stirred at room temperature (RT) for 24 h and ultrasonicated in a water bath for 15 min before use. 

An aqueous solution of poly (diallyldimethylammonium chloride) (PDDA) with a concentration of 20 wt% (an average molar mass of *M*_W_ = 200,000–350,000 g·mol^−^^1^, Sigma–Aldrich, Steinheim, Germany) was used. The stock solution of PDDA was prepared using MilliQ water at a concentration of 0.1 wt%. 

The Sap/PDDA organoclay dispersion was prepared by mixing the required volumes of Sap dispersion with the PDDA solution and MilliQ water. This mixture was heated in the water bath at 50 °C for 5 h. Subsequently, the colloidal dispersion of the prepared organoclay was cooled to RT. The concentration of Sap in the dispersion was 1 g L^−^^1^, and the loading of monomer units of PDDA (*n*_(PDDA)_/*m*_(Sap)_) was 1.5 mmol·g^–1,^ respectively. 

The Sap/PDDA/EryB dispersion was prepared by functionalization of Sap/PDDA dispersion with an aqueous solution of EryB (2 × 10^−^^4^ g L^−^^1^). The final concentration of Sap was 0.1 g L^−^^1^, the loading of monomer units of PDDA (*n*_(PDDA)_/*m*_(Sap)_) was 1.5 mmol·g^−^^1^ and the loading of EryB (*n*_(EryB)_/*m*_(Sap)_) was 1.5 mmol·g^−^^1^. This colloidal dispersion was stirred at room temperature for 72 h. PDDA was completely adsorbed onto the Sap particles, as the capacity of the silicate far exceeded the amount added. Of the EryB loading, 0.397 mmol g^−^^1^ was retained in the Sap/PDDA/EryB complex, as determined by analysis of the supernatants with non-adsorbed EryB. The EryB concentrations in the supernatants were analyzed by UV-Vis spectroscopy.

The nanocomposite discs (PU/Sap/PDDA or PU/Sap/PDDA/EryB, respectively) were prepared in two steps. In the first step, thin films of Sap/PDDA or Sap/PDDA/EryB were prepared by filtering 20 mL of the prepared dispersions (Sap/PDDA or Sap/PDDA/EryB) through a Teflon filter (pore size 0.1 µm, diameter 25 mm Omnipore, hydrophilic PTFE, Millipore, Merck, Darmstadt, Germany). The films were then washed with distilled water to remove non-adsorbed EryB and transferred to a Petri dish. In the second step, the layer of nanocomposite was immediately covered with a liquid PU precursor (0.013 mL cm^−^^2^) and left to dry and cure at RT in the flow of air. When the PU was dried, the Teflon filter was removed, and the final nanocomposite disc was obtained.

### 3.3. Characterization of Nanocomposites

Absorption spectroscopy in the UV−Vis region was measured using a double-beam Cary 5000 UV–Vis-NIR Spectrometer (Agilent, Santa Clara, CA, USA). The samples were measured using the Cary Universal Measurement accessory (UMA). Steady-state fluorescence measurements were performed using a Fluorolog-3 spectrometer (Horiba Jobin-Yvon, Kyoto, Japan) in the front-face setup. The samples were measured using the J1933 Solid Sample Holder from Horiba. The emission spectra were recorded upon excitation at 490 nm in the range from 500 to 850 nm. 

Attenuated total reflectance (ATR) infrared (IR) spectroscopy measurements were conducted on a Ni-colet™ is50 Fourier transform infrared (FTIR) spectrometer (Thermo Scientific, Waltham, MA, USA) equipped with a single-reflection ATR accessory with the diamond crystal. The spectra were recorded with a resolution of 4 cm^−^^1^ using the Thermo Scientific OMNIC™ 8.0 software. 

X-ray diffraction (XRD) patterns were recorded in reflection mode, using a fixed sample stage for flat samples, on the EMPYREAN system (PANalytical B.V. Westborough, MD, USA), equipped with CuKα (*λ*_α1_ = 1.54060 Å) radiation operating at 45 kV and 40 mA. The patterns were scanned in the 2*θ* range of 2.5–30° with scan steps of 0.026° 2*θ* and scan step times of 100 s.

### 3.4. Photosensitizing Properties of the Nanocomposites

DCFH DA (CAS 4091-99-0) was purchased from Merck (Darmstadt, Germany) and used as received. A 1 mM stock solution was prepared in DMSO (SERVA Elecrophoresis GmbH, Heidelberg, Germany). Then, 50 μL of the stock solution was mixed with 4.95 mL of 1 mM Na_2_HPO_4_ (AppliChem, Darmstadt, Germany) solution to hydrolyze acetate groups under the basic conditions. The reaction took 20 min with stirring in the dark, producing active DCFH. The solution was then neutralized with 5 mL of 1 mM NaH_2_PO_4_ (AppliChem, Darmstadt, Germany) solution and the freshly prepared DCFH probe was immediately used in the tests. The 1 cm × 1 cm squares of the specimens were transferred in 24 well microtiter plates, fixed on the well bottom with agarose (Promega, Madison, WI, USA) and covered with 1 mL of probe solution, then irradiated either with LED or laser, for 1.5 h or 10 min, respectively. The control samples of pure PU and the composite without EryB were also tested. The composite with EryB was tested also in the dark conditions to estimate the effect of irradiation. The formation of DCF as the product of the reaction of DCFH and ROS was analyzed using emission and fluorescence spectra. The wavelengths for emission (570 nm) and excitation (490 nm) were suitably selected to efficiently detect not only DCF, but also EryB molecules released from the tested materials.

### 3.5. Photodynamic Inactivation of Planktonic and Biofilms Cultures

A preincubated culture of *S. aureus* CCM 3953 was prepared according to the procedure described in Section 2.1 and a volume of 1 mL was transferred to the 24-well plate. This method for evaluating antibacterial activity was adopted from previous studies [36,37] with some modifications. The culture was centrifuged (Universal 32 R, Hettich Zentrifugen, Tuttlingen, Germany) for 10 min at 1840× *g* and at 15 °C. The medium was removed and 1 mL of the EryB solutions of 0.01 mmol L^−1^, 0.05 mmol L^−1^, or 0.1 mmol L^−1^ concentration was added to the wells. The plate was incubated on a thermoshaker (Thermostatic cabinet, Lovibonds, Biosan, Riga, Latvia) at 37 °C, 300 rpm, for 1 h, and then another centrifugation step was performed. The medium was aspirated, and the pellet was irradiated with a green laser or a green LED lamp at different periods. Subsequently, 1 mL of PBS was added to each well, followed by mixing and cell scraping to ensure uniform distribution. From the first well, 100 μL was transferred to the subsequent wells for further analysis. The bacterial suspension was then serially diluted using 900 μL of PBS. For plating, Müeller–Hinton agar (MHA, Biolife, Milan, Italy) was used. After incubation at 37 °C for 24 h, the colony-forming units (CFU mL^−^^1^) were calculated.

The 24 h biofilm was formed from the pre-incubation cultures prepared according to the procedure described in Section 2.1. A 24-well plate was used to incubate the biofilm. A volume of 0.5 mL of the pre-incubation culture and 0.5 mL of MHB were added to each well. The plates were incubated at 37 °C for 24 h, then tested with 1 mL of a 0.1 mmol L^−^^1^ and 0.05 mmol L^−^^1^ EryB solutions. The concentration of PS was selected after the results of the tests with planktonic cultures. Incubation was performed in the dark at 37 °C for 1 h. To compare the anti-biofilm effect, the cultures were irradiated with a green laser for 10 min and a green LED light for 1.5 h. The biofilm was scraped from the bottom of the plates, serially diluted in PBS, and plated on MHA. Calculation of CFU mL^−^^1^ was determined after 24 h. 

Results from both experiments with planktonic culture and biofilm were calculated from 3 minimal parallel wells and results represent the average with standard error. Experiments were repeated for minimal three times.

### 3.6. PDI of S. aureus Biofilm Formed on Nanocomposite

**Figure 7:**
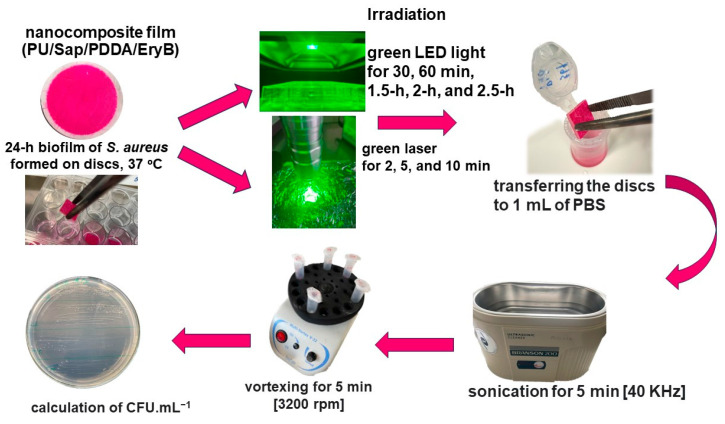
Schematic assay of testing antimicrobial activity on nanocomposites.

PU discs coated with composite films were cut into squares (1 cm × 1 cm) and sterilized by UV irradiation for 10 min on both sides [36]. Antimicrobial testing is briefly summarized in Figure 7, briefly; biofilm growth was initiated in 24-well microtiter plates. A pre-incubated culture of CCM 3953 (OD_600_ = 0.5) was added to the wells (0.5 mL), along with 0.5 mL of MHB. The plates were then incubated at 37 °C for 24 h to facilitate biofilm formation. The impact of irradiation on nanocomposite samples containing EryB (Sap/PDDA/EryB) was examined using a green laser for 2 min, 5 min, 10 min, and with green LED light for 30 min, 60 min, 1.5 h, 2 h, and 2.5 h. Subsequently, all samples were immersed in 1 mL of PBS, sonicated, vortexed (Vortex V-1 plus, Biosan, Riga, Latvia), serially diluted, and plated on MHA plates. CFU mL^−^^1^ was determined after 24 h. Each sample was tested in at least five parallel runs. The results are given as mean values and standard deviations.

Selected samples of collected biofilm cells (from PU alone; SAP/PDDA; SAP/PDDA/EryB; nano + LED 1.5 h; nano + laser 10 min) were observed in a fluorescence microscopy (Intraco Micro LM 600, Tachlovice, Czech Republic). Cells were pooled from three discs per each sample and resuspended in 100 μL of PBS. The volume of 20 μL of the suspension was taken into an Eppendorf tube for staining with 4 μL of DAPI (0.5 mg/mL; 4′,6-diamidino-2-phenylindole, Sigma Aldrich, Darmstadt, Germany) for 1 min. Then, 10 μL of each sample was pipetted on a glass slide, covered with a cover glass with a drop of immerse oil, and immediately observed (Immersol™ Immersion Oil, Zeiss, Germany; magnification 1000-fold).

### 3.7. Statistical Analysis

Statistical significance was determined with the two-tailed Student’s *t*-test GraphPad Prism using software (Graph Pad, San Diego, CA, USA). *p*-value < 0.05 (*) was considered statistically significant; *p* < 0.01 (**); *p* < 0.001 (***); *p* < 0.0001 (****).

## 4. Conclusions

This study demonstrates the promising potential of PDI using the photosensitizer EryB integrated into PU-based nanocomposites to combat *S. aureus* biofilm. The integration of EryB on the surface of modified silicate particles (Sap) and into the PU nanocomposite matrix did not significantly alter the spectral properties or photoactivity of the dye, as confirmed by visible light absorption and fluorescence spectroscopy. This indicates that EryB retains its functional properties within the composite material. The enlargement of the interlayer spaces of the silicate demonstrated by X-ray diffraction supports the successful integration of the components, polycation, EryB, and PU. Infrared spectroscopy also confirmed the presence and interaction of the individual components within the nanocomposite. The experimental results show that EryB solutions exhibited a significant antibacterial effect when irradiated with both LED light and green laser. EryB solutions of concentrations of 0.05 mmol L^−^^1^ and 0.1 mmol L^−^^1^ irradiated with green LED light for 1.5 h or a green laser for 10 min were able to reduce bacterial growth in planktonic cells and biofilms by up to 10,000-fold. These results were confirmed in experiments with nanocomposites and highlight the effectiveness of PDI in disrupting *S. aureus* biofilms, resulting in a strong bactericidal effect. The results with control samples or without irradiation confirm the crucial role of PDI. The photosensitizing properties of the composite and EryB release from the material were confirmed by the experiments using the ROS-sensitive probe. As antibiotic resistance is a major challenge, the results of this work provide further evidence that PDI is a viable alternative to conventional treatment. Future research could focus on optimizing the concentration and irradiation parameters, exploring other photosensitizers, engineering polymers, or modification designs, and testing the long-term stability and efficacy of these nanocomposite materials in a clinical setting. This approach is in line with the ongoing search for innovative antimicrobial strategies and could lead to the development of new medical materials and devices that can effectively prevent and control forming bacterial biofilms.

## Data Availability

Data are available on request.

## Supplementary Materials

The following supporting information can be downloaded at: https://www.mdpi.com/article/10.3390/molecules29163917/s1, Figure S1: The anti-biofilm effectiveness of two different concentrations of EryB against *S. aureus* CCM 3953. PDI was performed with a green LED light (1.5 h) and a green laser (10 min); Figure S2: The spectrum of LED light source.
