# Supplementary material for: Anti-Biofilm Effect of Hybrid Nanocomposite Functionalized with Erythrosine B on Staphylococcus aureus Due to Photodynamic Inactivation
Source: Molecules. 2024 Aug 19;29(16):3917. doi: 10.3390/molecules29163917 (PMC11357139; doi:10.3390/molecules29163917)
Supplement: Supplementary file 1 [file molecules-29-03917-s001.zip › molecules-3121291-supplementary.pdf]

Supplementary data

Anti-Biofilm Effect of Hybrid Nanocomposite Functionalized with Erythrosine B on *Staphylococcus aureus* Due to Photodynamic Inactivation

Larysa Bugyna[1], Katarína Bilská[1], Peter Boháč[3], Marek Pribus[3], Juraj Bujdák[2,3] and Helena Bujdáková[1*]

[1] Department of Microbiology and Virology, Faculty of Natural Sciences, Comenius University in Bratislava,
Ilkovičova 6, 842 15 Bratislava, Slovakia; larysa.bugyna@uniba.sk (L.B.); bilska6@uniba.sk (K.B.)
[2] Department of Physical and Theoretical Chemistry, Faculty of Natural Sciences,
Comenius University in Bratislava, Ilkovičova 6, 842 15 Bratislava, Slovakia; juraj.bujdak@uniba.sk (J.B.)
[3] Institute of Inorganic Chemistry, Slovak Academy of Sciences, Dúbravská Cesta 9, 845 36 Bratislava, Slovakia;
peter.bohac@savba.sk (P.B.); marek.pribus@savba.sk (M.P.)
* Correspondence: helena.bujdakova@uniba.sk (H.B.*)

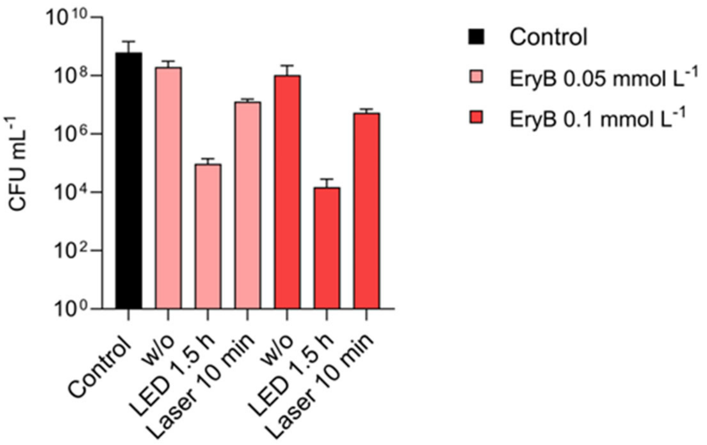

Figure S1. The anti-biofilm effectiveness of two different concentrations of EryB against *S. aureus* CCM 3953. PDI was performed with a green LED light (1.5 h) and a green laser (10 min).

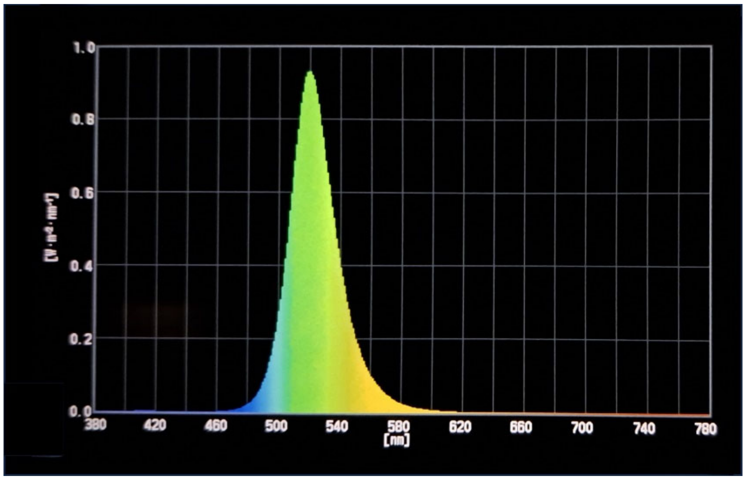

Figure S2. The spectrum of LED light source.
